# Alcohol Text Messages: A Developmental Study

**DOI:** 10.1007/s11469-017-9835-y

**Published:** 2017-11-28

**Authors:** Maryanne Robinson, R. B. Wilkinson, R. Fletcher, R. Bruno, A. L. Baker, L. Maher, J. Wroe, A. J. Dunlop

**Affiliations:** 1 School of Medicine and Public Health, Faculty of Health and Medicine, University of Newcastle, Callaghan, New South Wales Australia; 2 12 Alfred Street, Newcastle East, New South Wales 2300 Australia; 3 School of Psychology, Faculty of Science, University of Newcastle, Callaghan, New South Wales Australia; 4 Family Action Centre, School of Medicine and Public Health, Faculty of Health and Medicine, University of Newcastle, Callaghan, New South Wales Australia; 5 School of Medicine, University of Tasmania, Hobart, Australia; 6 The Kirby Institute, University of New South Wales, Sydney, New South Wales Australia; 7 Drug and Alcohol Clinical Services, Hunter New England Local Health District, New Lambton, New South Wales Australia; 8 Centre for Brain and Mental Health, University of Newcastle, Callaghan, New South Wales Australia; 9 Hunter Medical Research Institute, New Lambton Heights, New South Wales Australia

**Keywords:** Alcohol, Fathers, Pregnancy, Messages, Cell phones

## Abstract

Risky paternal alcohol use is associated with maternal alcohol use during pregnancy, poor fetal and infant outcomes, domestic violence and depression. This study developed 30 SMS text messages about alcohol for fathers who drink at risky levels*.* The text messages were developed using two motivational styles: messages presented in a second person voice and the same messages presented in a child’s voice. Fifty-one fathers were recruited through social media to complete an online survey rating the SMS text messages for message importance and likelihood of seeking further information and measuring risky alcohol use and psychosocial distress. Seventeen participants then participated in a semi-structured qualitative interview. Fathers rated the text messages presented in the child’s voice as more important than messages presented in the second person. Qualitative data supported survey results that motivational SMS text messages could provide an acceptable way to raise awareness of risky alcohol consumption for future fathers.

Information regarding alcohol-related harm during the perinatal period has been directed to prospective mothers but seldom to their male partners (Elliot et al. 2006; Elliott 2014; McBride 2014). This is of concern, as paternal alcohol consumption is associated with poor infant outcomes including increased risk of fetal alcohol spectrum disorders (FASD), reduced infant birth weight, cognitive impairment and congenital heart defects (Abel 2004). Fetal alcohol spectrum disorders are the leading cause of preventable environmental birth defects and intellectual disability in Australia and occur as a direct result of alcohol exposure in pregnancy (Elliott 2014). Harmful paternal alcohol use is also implicated in child maltreatment, family breakdown and domestic violence (Laslett et al. 2015).

A recent review of the effects of parental alcohol consumption on their offspring showed that, in studies where prevalence of alcohol use was recorded, between 77 and 96% of men consumed alcohol throughout the pregnancy (McBride and Johnson 2016). This review explored the role of fathers’ alcohol consumption on their offspring and found evidence across 11 studies that men who drink alcohol before conception and during their partner’s pregnancy may increase the chance of their partner drinking alcohol, negatively impact the quality of their relationship, decrease sperm quality and increase the risk of spontaneous abortion as well as other possible negative outcomes (McBride and Johnson 2016).

Alcohol consumption during pregnancy remains a major public health problem and is associated with multiple harmful maternal and fetal effects (Forray and Foster 2015). The safe lower limit of alcohol consumption during pregnancy has not been established (Cohn et al. 2011), and the prevalence of alcohol use in pregnancy varies. In Australia, up to half of all pregnant women report consuming any alcohol during pregnancy (Australian Institute of Health and Welfare 2016). In the USA, 30% of women consume alcohol during pregnancy (Ethen et al. 2009) while in European countries, between 5 and 60% of pregnant women drink alcohol (Skagerström et al. 2013). Strategies involving reducing alcohol use by male partners may be an effective way to reduce maternal alcohol consumption.

Interventions using short message service (SMS) text messaging could be an effective way to reduce risky drinking (Kaner et al. 2015; Kypri et al. 2003; Remick and Kendrick 2013). Mobile phone technology has been suggested as a possible way to deliver interventions regarding alcohol (Cohn et al. 2011). The benefits described include anonymity when disclosing excessive alcohol consumption and the low cost of providing the intervention, as well as the ability to contact hard-to-reach target populations not currently in contact with health care services.

Importantly, a personalised telephone SMS project targeting at-risk mothers in the USA was effective in reducing barriers to accessing health and safety information about pregnancy and a baby’s first year of life (text4baby) (Remick and Kendrick 2013). The US Centres for Disease Control and Prevention developed the messages in accordance with national guidelines then tested the messages on expectant and new mothers to gain feedback (Whittaker et al. 2012). This free health text messaging service reached over 320,000 women in 2012 and delivered many of the messages in a ‘child’s’ voice (as though the child was speaking directly to the mother) as a means of effecting behaviour change. Prenatal and postnatal messages included topics on smoking, alcohol and other drugs and how to access related health care using toll-free numbers.

While text messages have previously been developed to provide health information for mothers during the perinatal period (Whittaker et al. 2012), and alcohol text messages have been developed to reduce alcohol related harm (Kaner et al. 2015; Sharpe et al. 2015), to our knowledge, this is the first study to develop and evaluate alcohol text messages for new and expectant fathers. SMS4dads is currently running as a study in Australia, providing support and information regarding mental health to new fathers’ mobile phones (Fletcher et al. 2017). The alcohol text messages for the current study were developed so they could be later incorporated into this larger study.

The current study aimed to develop 30 text messages to inform men about the benefits of reducing risky drinking during the transition to fatherhood. We wanted to pilot the messages with experienced fathers to see what messages about alcohol they considered important for new fathers to receive. More specifically, we wanted to explore whether messages that used the ‘child’s voice’ increased the base rate of positive responses compared to messages presented in the second person.

Based on the presumption that text messages in the child voice would be more salient, we predicted that fathers would rate the alcohol text messages presented in the child’s voice as more important than alcohol text messages presented in a second person voice. We anticipated this effect would be more pronounced in fathers who drink at risky levels and in those with more psychological distress. We also anticipated that fathers would be more likely to seek additional information for alcohol text messages presented using the child’s voice compared to messages in a second person voice. We predicted this effect to be greater in fathers who drink at risky levels and in fathers with more psychological distress. Finally, we predicted that fathers’ ratings of message importance would influence the likelihood of them pressing on a hypothetical link for additional information.

## Method

### Participants

A convenience sample of 51 adult men (> age 18 years) was recruited through social media (Facebook, Twitter and LinkedIn) from June through July 2016. Entry criteria included being a current or expectant Australian father, having ever consumed alcohol and having access to the Internet. Participants were entered into a draw to receive a $100 store gift voucher after completing the survey. Ethical approval for the study was granted by the University of Newcastle, Australia Human Research Ethics Committee, approval number H-2016-0136.

### Procedure

Thirty short messages (< 160 characters) were developed by the first author (MR). The messages addressed alcohol-related harms in relation to infant health, the spousal relationship, father-infant relationship and the fathers’ health. Messages were based on national alcohol guidelines (National Health and Medical Research Council 2009) regarding alcohol consumption. Motivational Interviewing (MI) techniques were considered in the construction of the text message language (Miller and Rollnick 2012), with a primary focus of encouraging self-reflection (Miller and Rollnick 2012). The Stages of Change model (Prochaska and DiClemente 1983; Prochaska et al. 2013) underpinned the text message design. This model focuses on self-directed intentional change, as opposed to imposed change for the modification of substance use (DiClemente 1993), and has proved effective in treating substance use disorders for over 30 years (Prochaska et al. 2013). Individuals are thought to move through a series of stages: precontemplation, contemplation, preparation, action and maintenance when changing unhealthy behaviours. Providing information about alcohol-related harm during the perinatal period may prompt some fathers to contemplate reducing the amount they drink or take action by ceasing alcohol. Information-seeking behaviour (by pressing on a hypothetical hypertext link relating to a particular text topic) indicates a proactive response according to this model.

Messages were refined using a modified Delphi system (Dalkey and Helmer 1963) with feedback from co-authors (ALB, AJD) and an alcohol researcher at the University of Newcastle. The text messages were presented using two types of message voice: a child’s voice, as if the father’s own child were addressing him e.g. ‘Hey dad, do you know how many drinks you can have before it affects your health?’ or a second person voice e.g. ‘Do you know how many drinks you can have before it affects your health?’ The messages had a Flesch-Kincaid grade level of 5.4 to ensure ease of comprehension.

All participants viewed and rated the same 30 messages during an on-line survey using the Qualtrics platform (Qualtrics software 2015). Responses were entered by participants via smart phone, tablet or computer into the on-line survey. The messages were not sent to participants’ mobile phones but rather were developed so they could be sent as SMS text messages in future research. Participants rated messages for importance using two 3-point Likert scales (*Important*, *Can’t Say*, *Not Important*) and whether participants would use a hypothetical hypertext link for more information on the message topic (*Likely*, *Can’t say*, *Unlikely*) (active links were not inserted). The order of the SMS text messages was randomised for each participant using an inbuilt randomisation feature of the program. The text message responses were then coded into one of two categorical variables: For importance, 0 = ‘not important’ or ‘can’t say’ and 1 = ‘important’, and for ‘likelihood’ of seeking further information, 0 = ‘not likely’ or ‘can’t say’ and 1 = ‘likely’. This produced a continuous variable for importance of the second person voice (0–15) and child voice (0–15), summed together for a total importance score (0–30), and a continuous variable for likelihood of seeking further information for the second person voice (0–15) and child voice (0–15) summed together for a total likelihood score (0–30).

National Health and Medical Research Council Guidelines for alcohol consumption (National Health and Medical Research Council 2009) were used in the design of the SMS text messages. Alcohol guidelines one to four regarding recommended alcohol consumption were presented to fathers in the survey before they answered the two final questions in the on-line survey. Fathers self-rated the importance and difficulty of drinking alcohol at levels recommended to reduce short-term and long-term harm based on the Australian alcohol guidelines using two 10-point Likert scales (with 1 equal to not important through to 10 being extremely important, and 1 being equal to not at all difficult through to 10 being very difficult to drink at recommended levels).

### Data Analysis

Data were analysed using SPSS version 22 software (IBM Software 2016). Analyses were performed using a 2 × 2 × 2 mixed ANOVA design. The first factor was a within-subjects factor of voice, with two levels, child voice or a second person voice. The second factor was a between-subjects factor of ‘at-risk’ alcohol consumption, with two levels, operationalized by summing self-reported AUDIT C scores (Bush et al. 1998) as low risk (0–3) or high risk (4–12). The third factor was a between-subjects factor of psychological distress, with two levels, operationalized by summing self-reported Kessler-6 (Kessler et al. 2002) scores as low distress (6–12) and moderate (13–18) to high distress (19–30). The moderate and high distress groups were collapsed into one group due to the small sample size.

After completing the on-line survey, participants were asked if they were interested in being contacted by telephone for a brief qualitative interview (*N* = 17) Data were transcribed verbatim and pseudonyms were assigned prior to coding using Template Analysis (King 1998). A priori themes included the acceptability of the messages, the importance of the alcohol messages, receptiveness to the design of messages in second person and child voice format and willingness to seek additional information. Themes were identified, coded and summarised into a template.

## Results

The majority of participants were current fathers (96%) in a relationship (86%), who had completed or commenced a tertiary education (77%), and were employed (98%) (Table 1).

**Table 1.** Demographic characteristics of participants

Variable	*n* (%)
Parenting experience	
Expectant father	2 (3.9)
Current father	49 (96.1)
Living arrangements	
Married/de facto	44 (86.3)
Separated	7 (13.7)
Age group	
25–35 years	13 (25.5)
36–50 years	38 (74.5)
Education level	
Year 10 or below	2 (3.9)
Years 11 or 12, TAFE	10 (27.4)
University under/postgraduate	39 (76.5)
Employment	
Full-time/part-time	50 (98.0)
Unemployed	1 (2.0)

Most fathers’ (65%) current alcohol levels were considered risky according to the Australian Alcohol Guidelines (Table 2). Based on the Audit C, around a third of fathers reported risky alcohol use on a monthly basis (29%), a quarter reported risky drinking some days a week (25%) and another quarter reported risky drinking less than monthly (25%), while 18% of men reported no risky alcohol use. The majority of participants reported no psychological distress (64%).

**Table 2.** Alcohol and psychological distress measures

		Factor scores	
Variable	*n* (%)	*M*	SD
AUDIT C scores	51 (100)	5.37	2.39
Low risk (0–4)	18 (35.3)	2.72	0.96
At risk (5–12)	33 (64.7)	6.82	1.53
Kessler 6 score	51 (100)	10.51	3.88
No psychological distress (6–12)	33 (63.7)	8.24	1.84
Medium psychological distress (13–18)	15 (29.4)	13.47	1.30
High psychological distress (19–24)	3 (5.8)	20.67	2.08
Motivational Interviewing ruler—importance	51 (100)	7.57	2.05
Motivational Interviewing ruler—difficulty	51 (100)	5.08	2.67

Participants rated the 30 messages about alcohol as being important overall (*M* = 22.00, SD = 6.83; possible total score 0–30). This included the 15 alcohol messages in the child voice (*M* = 11.81, SD = 4.07) (Table 3 possible score 0-15) and 15 messages in a second person voice (*M* = 10.60, SD = 3.80) (Table 4, possible score 0–15). A higher rating of importance was given to messages in the child voice (*M* = 11.81) compared to those in a second person voice (*M* = 10.60), *F*(1, 47) = 15.44, *p* < 0.001, partial *η* ^2^ = .247, Hedges’ *g* = 0.3. There were no other main effects or interactions that approached statistical significance (all *p* > 0.16).

**Table 3.** Text messages ranked by importance—child voice

	Child voice ranking *n* (%)		
Text message	Important	Can’t say	Not important
Hey dad, did you know drinking less alcohol now safeguards me from developing behavioural problems when I'm older?	47 (92.2)	4 (7.8)	0 (0.0)
Hey dad, your going to need all the sleep you can get. It might surprise you, drinking alcohol worsens your sleep	45 (88.2)	6 (11.8)	0 (0.0)
Hey dad, did you know that drinking alcohol daily can cause health problems. Want to find out more?	44 (86.3)	5 (9.8)	2 (3.9)
Hi dad, do you know how many drinks you can have before it harms your health? Want to know more?	43 (84.3)	5 (9.8)	3 (9.8)
Hey dad, did you know if you drink less you will bond better with me. Want to know more?	42 (82.4)	8 (15.7)	1 (2.0)
Hey dad, did you know Fetal Alcohol Spectrum Disorders are the leading cause of preventable birth defects?	42 (82.4)	7 (13.7)	2 (3.9)
Hey dad, keeping your drinking down keeps our family strong.	42 (82.4)	7 (13.7)	2 (3.9)
Hi dad, having trouble finding the energy to get going? Ever wondered if drinking might be taking it out of you?	41 (80.4)	8 (15.7)	2 (3.9)
Hi dad, want to quit worrying so much? Did you know cutting down on alcohol helps?	40 (78.4)	6 (11.8)	5 (9.8)
Sometimes dad, sometimes just reducing alcohol helps to reduce your stress. Ever thought that cutting back a bit might be something worth doing?	40 (78.4)	9 (17.7)	2 (3.9)
Hi dad, having trouble concentrating? Did you know drinking can make it hard to think clearly?	40 (78.4)	9 (17.7)	2 (3.9)
Want to be there for me when I grow up, Dad? Did you know heavy drinking can increase your chances of injury and accidents	38 (74.5)	8 (15.7)	5 (9.8)
Hey dad, want to support mum by taking a break from alcohol? Lots of well known dads are doing it	33 (64.7)	14 (27.5)	4 (7.8)
Hi dad, did you know alcohol can really upset your digestion?	29 (56.9)	9 (17.7)	13 (25.5)
Sore head from drinking dad? Want to know how to lose those headaches?	23 (45.1)	13 (25.5)	15 (29.4)

**Table 4.** Text messages ranked by importance—second person voice

	Generic voice ranking *n* (%)		
Text message	Important	Can’t say	Not important
Did you know drinking less alcohol now safeguards me from developing behavioural problems when I'm older?	47 (92.2)	4 (7.8)	0 (0.0)
You’re going to need all the sleep you can get. It might surprise you, drinking alcohol worsens your sleep	43 (84.3)	5 (9.8)	3 (5.9)
Did you know that drinking alcohol daily can cause health problems. Want to find out more?	41 (80.4)	6 (11.8)	4 (7.8)
Do you know how many drinks you can have before it harms your health? Want to know more?	41 (80.4)	7 (13.7)	3 (5.9)
Did you know if you drink less you will bond better with me. Want to know more?	42 (82.4)	8 (15.7)	1 (2.0)
Did you know Fetal Alcohol Spectrum Disorders are the leading cause of preventable birth defects?	45 (88.2)	5 (9.8)	1 (2.0)
Keeping your drinking down keeps our family strong.	31 (60.8)	14 (27.5)	6 (11.8)
Having trouble finding the energy to get going? Ever wondered if drinking might be taking it out of you?	36 (70.6)	10 (19.6)	5 (9.8)
Want to quit worrying so much? Did you know cutting down on alcohol helps?	35 (68.6)	11 (21.6)	5 (3.9)
Sometimes just reducing alcohol helps to reduce your stress. Ever thought that cutting back a bit might be something worth doing?	42 (82.4)	6 (11.8)	3 (5.9)
Having trouble concentrating? Did you know drinking can make it hard to think clearly?	37 (72.6)	11 (21.6)	3 (5.9)
Want to be there for your child when they grow up? Did you know heavy drinking can increase your chances of injury and accidents	41 (80.4)	4 (7.8)	6 (11.8)
Want to support mum by taking a break from alcohol? Lots of well known dads are doing it	31 (60.8)	14 (27.5)	6 (11.8)
Did you know alcohol can really upset your digestion?	24 (47.1)	12 (23.5)	15 (29.4)
Sore head from drinking? Want to know how to lose those headaches?	25 (49.0)	9 (17.7)	17 (33.3)

In terms of additional information seeking assessed by the likelihood of pressing a hypothetical hypertext link (Tables 5 and 6), fewer items were rated overall as likely to prompt information seeking (*M* = 14.57, SD = 9.15; total possible score 0–30). There were similar ratings of likelihood of pursuing information between child voice (*M* = 7.51, SD = 5.05; Table 5 possible score 0–15) and second person voice (*M* = 7.49, SD = 5.27; Table 6, possible score 0–15). There were no significant differences in the importance of child voice compared to second person voice on sub-group analyses.

**Table 5.** Text messages ranked by likelihood—child voice

	Child voice ranking *n* (%)		
Text message	Likely	Can’t say	Not likely
Hey dad did you know drinking less alcohol now safeguards me from developing behavioural problems when I'm older?	38 (74.5)	5 (9.8)	8 (15.7)
Hi dad, do you know how many drinks you can have before it harms your health? Want to know more?	35 (68.6)	7 (13.7)	9 (17.7)
Hey dad, did you know if you drink less you will bond better with me? Want to know more	34 (66.7)	8 (15.7)	9 (17.7)
Hey dad, did you know Fetal Alcohol Spectrum disorders are the leading cause of preventable birth defects?	32 (62.8)	10 (19.6)	9 (17.7)
Hey dad, your going to need all the sleep you can get. It might surprise you, drinking alcohol worsens your sleep	31 (60.8)	10 (19.6)	10 (19.6)
Hey Dad, want to support mum by taking a break from alcohol? Lots of well known dads are doing it	27 (52.9)	11 (21.6)	13 (25.5)
Hey dad, did you know that drinking alcohol daily can cause health problems. Want to find out more?	26 (51.0)	13 (25.5)	12 (23.5)
Hi dad, having trouble finding the energy to get going? Ever wondered if drinking might be taking it out of you?	24 (47.1)	12(23.5)	15 (29.4)
Hi dad, want to quit worrying so much? Did you know cutting down on alcohol helps?	24 (47.1)	11 (21.6)	16 (31.4)
Sometimes dad, just reducing alcohol helps to reduce your stress. Ever thought that cutting back a bit might be something worth doing?	22 (43.1)	14 (27.4)	15 (29.4)
Hey dad, keeping your drinking down keeps our family strong.	22 (43.1)	12 (23.5)	17 (33.3)
Hi dad, having trouble concentrating? Did you know drinking can make it hard to think clearly?	21 (41.2)	17 (33.3)	13 (25.5)
Want to be there for me when I grow up, Dad? Did you know heavy drinking can increase your chances of injury and accidents	18 (35.3)	12 (23.5)	21 (25.5)
Hi dad, did you know alcohol can really upset your digestion?	16 (31.4)	13 (25.5)	22 (43.1)
Sore head from drinking dad? Want to know how to lose those headaches?	13 (25.5)	13 (25.5)	25 (43.1)

**Table 6.** Text messages ranked by likelihood—second person voice

	Generic voice ranking *n* (%)		
Text message	Likely	Can’t say	Not Likely
Did you know drinking less alcohol now safeguards me from developing behavioural problems when I'm older?	34 (66.7)	10 (19.2)	7 (13.7)
You’re going to need all the sleep you can get. It might surprise you, drinking alcohol worsens your sleep	35 (68.6)	5 (9.8)	11 (21.6)
Did you know that drinking alcohol daily can cause health problems. Want to find out more?	34 (66.7)	8 (15.7)	9 (17.8)
Do you know how many drinks you can have before it harms your health? Want to know more?	34 (66.7)	9 (17.7)	8 (15.7)
Did you know if you drink less you will bond better with me. Want to know more?	25 (49.0)	12 (23.5)	14 (29.4)
Did you know Fetal Alcohol Spectrum Disorders are the leading cause of preventable birth defects?	21 (41.2)	13 (25.5)	17 (33.3)
Keeping your drinking down keeps our family strong.	23 (45.1)	9 (17.7)	19 (37.3)
Having trouble finding the energy to get going? Ever wondered if drinking might be taking it out of you?	23 (45.1)	12 (23.5)	16 (31.4)
Want to quit worrying so much? Did you know cutting down on alcohol helps?	22 (43.1)	14 (27.5)	15 (29.4)
Sometimes just reducing alcohol helps to reduce your stress. Ever thought that cutting back a bit might be something worth doing?	23 (45.1)	9 (17.7)	19 (37.3)
Having trouble concentrating? Did you know drinking can make it hard to think clearly?	21 (41.2)	13 (25.5)	17 (33.3)
Want to be there for your child when they grow up? Did you know heavy drinking can increase your chances of injury and accidents	20 (39.2)	17 (33.3)	14 (27.5)
Want to support mum by taking a break from alcohol? Lots of well known dads are doing it	18 (35.3)	8 (15.7)	25 (49.0)
Did you know alcohol can really upset your digestion?	19 (37.3)	12 (23.5)	20 (39.2)
Sore head from drinking? Want to know how to lose those headaches?	16 (31.4)	11 (21.6)	24 (47.1)

Using the motivational rulers, on self-assessment of adhering to local alcohol guidelines, participants felt it was reasonably important to adhere to guidelines (*M* = 7.57, SD = 2.07) and rated this as moderately difficult to do (*M* = 5.08, SD = 2.67). Participants who consumed alcohol at high-risk levels rated importance of drinking to recommended guidelines as less important than those who drank at low-risk levels. There was a significant negative correlation between Audit C score and self-rating of importance of drinking according to alcohol guidelines, (*r* = − .370, *N* = 51, *p* = .008, two-tailed). Participants who consumed alcohol at high-risk levels found it more difficult to drink according to guideline recommendations than those who drank at low-risk levels. There was a significant positive correlation between Audit C score and self-rated difficulty of drinking according to Australian Guidelines, (*r* = .560, *N* = 51, *p* < .001, two-tailed).

### Qualitative Analysis

Seventeen participants (17/51, 33%) who agreed to be contacted for a telephone interview included both younger fathers (8/17) and older fathers (9/17). The representation of participants who consumed alcohol at high-risk levels (10/17, 59%) was similar to the quantitative sample of high-risk drinkers (65%). The proportion of low-risk drinkers (6/17) were evenly spread between the age groups. The final participant was a younger father who consumed alcohol at high levels when his first child was born but was now an ex-drinker.

Most interview participants responded positively to the text messages and noted they found them to be non-judgmental in style. Jim, an older father and risky drinker said, ‘I didn’t find them preachy. I thought they were straight up statements’. Craig, a younger father and risky drinker said, ‘I like the way it did not imply you have a drinking problem’. Alex, a younger father and low-risk drinker, also indicated acceptability and potential benefit: ‘I will be absolutely honest …for my first child, I probably needed them’. However, one participant, John, a younger father and risky drinker, did not like the tone of the messages stating: ‘I found that the messages were quite condescending’. The potential impact of a text messaging intervention for fathers was highlighted by Ryan: ‘For a number of years I was a very heavy drinker… at that time having an objective external voice prompting me to think about my choices would have been really useful.’

Participants also consistently indicated that they thought the alcohol text messages were important. The messages rated as most important among participants, included the effects of paternal alcohol consumption on paternal bonding, sleep deprivation, developmental problems, behavioural issues and FASD. Participants repeatedly stated they thought messages that directly connected the father’s behaviour with their child’s health and emotional wellbeing had greater impact than the messages about their own health. For example, Craig a younger father and risky drinker, said:

I would try and tailor it more to the relationship - like the ones purely about your health needs to be related back to your relationship with the baby. Especially if you are targeting the niche market for fathers where the emotion is fresh … like the one about bonding, like the ones about how alcohol affects your family.

Cameron, an older father and risky drinker, felt that ‘the ones that discuss the importance of connecting on an emotional level with your child’ were particularly important for him. Kelvin, an older father and risky drinker, said ‘It is really important to know that alcohol can damage the baby and this could bring awareness to men about fatherhood…you know link the two up.’

The effects of drinking on sleep and ability to cope with stress were frequently raised as issues for new fathers. As Marc, an older father and low-risk drinker, said:

I think the things that struck me as particularly useful were … regular and or heavy alcohol use can negatively affect your sleep, coping with stress, energy levels … most blokes I know still think that alcohol helps them sleep, cope with stress… I think the big one is the sleep deprivation you and mum go through for the first year or two with each child. I think that is a big selling point for change in alcohol use.

Interestingly, the ten participants interviewed with at-risk AUDIT-C scores who responded to the qualitative survey provided positive feedback about the messages. Jim, an older father who scored in the high range on both the AUDIT-C (8) and the K6 (20) said, ‘Look I probably scored every single one of those statements as important’. He then went on to say they were ‘sort of confronting …like the statements about the impact of the drinking on the child in a sort of future sense’ and that made the messages ‘…more important in a sense’.

Further, participants reported that messages using the child’s voice made them aware of their paternal responsibilities and how their drinking behaviour may impact upon their child. For example, Jerome, a younger low-risk drinker, said the child voice ‘makes you think there is another person involved… makes it concrete... I think it reminds you are dealing with a person’. Further support for salience of the child’s voice came from Ryan who said, “I thought for me it felt like… this even makes me feel more intensely’. Similarly, Kelvin indicated ‘The ones from the child helped make you think that there was someone else involved. It made you think about someone other than yourself and that you are affecting. I think it worked better than the other messages’. Darren, an older father and low-risk drinker suggested ‘I think that the ‘hey dad’ would work for new fathers especially because they are not used to being called dad yet. I think that could add something extra’. The fathers who previously drank heavily were generally more enthusiastic about the potential of the child’s voice to raise awareness about the impact of their alcohol consumption.

Importantly and in contrast to the quantitative data, the qualitative data also indicated potential for the child’s voice to be seen as manipulative and ineffectual. A minority of participants expressed concerns about feeling manipulated. For example, Marc, an older father and risky drinker, who generally approved of the messages, had an issue with the child voice:

It is probably just me, still with some vestigial oppositional tendencies, but I found the messages with the so called ‘child voice’ got my back up a bit, it almost seemed like a form of emotional blackmail… trying to get at me through my baby… and I felt like I would not buy into it by responding to any of them. Whereas the messages directed to me as one adult to another seemed very reasonable and I was happy to take them on board.

Another area explored during the interview was whether the messages made participants more likely to seek more information. A common theme expressed by participants was that they liked the format of brief messages with the option of receiving further information. For example, Craig said ‘I have always been a fan of if I receive something in communication you’ve got a really short brief message, a snap shot, and if you want more information you go and get more’. Most of the fathers indicated that they would access information if it were directly linked to fatherhood and developmental concerns. For example, as noted by Darren:

I like the ones that link the fathers to the kids in developmental studies. If you go on a website there is heaps of information for mothers but not a huge amount for men, so if you could sort of say one of the benefits of you drinking less, it’s been shown in this study to have this affect that would be pretty interesting.

When fathers were asked for their own ideas regarding alcohol text messages they thought were most important to send to future fathers, most expressed support for messages about reducing excessive paternal alcohol consumption. Interestingly, ex-drinkers and risky drinkers indicated a preference for the strongly worded alcohol messages. Ryan suggested, ‘Is this the person you want to be? Is this the parent you would want to be?’ Jim suggested, ‘Have you thought about how many drinks you’re going to have tonight? It’s not impossible to stop at two!’ Another participant highlighted the importance of paternal health in the post-partum period. For example, Marc suggested: ‘New dads aren’t getting enough sleep, feel tired all the time, and certainly don’t want to do anything that makes it worse’. Aiden, an older father and risky drinker had numerous suggestions including self-health messages ‘Did you realise more than 3-4 drinks a day is considered excessive drinking?’ Suggested text messages also focused on fathers’ psychological health. For example, Jim suggested: ‘Did you know that excessive drinking can be isolating and lead to depression?’

Text messages concerning the effects of drinking alcohol on family life were also offered by a small group of fathers, like Aiden, ‘An hour spent drinking after work is an hour you could have played backyard cricket with your children’. Marc went further by highlighting the importance of prenatal health for fathers: ‘Getting healthy before conception is important… Women have been on to this for a long time’. These text message suggestions highlight the alcohol-related harms that this group of fathers thought were important to raise awareness of in new fathers. These data also demonstrate that the fathers who participated in the study were reflecting on alcohol-related harm and how it impacted their parenting. Many participants openly discussed how the text messages made them reflect on their own drinking.

## Discussion

The current study developed and piloted a bank of alcohol text messages that have the potential to reduce alcohol-related harms in prospective and new fathers. Fathers rated alcohol text messages presented in the child’s voice as more important than alcohol text messages that used a second person voice. This is an interesting finding given the small sample size and is an area that warrants further investigation to better understand the role of text messages in alcohol behaviour change for new fathers. The small sample size of the current study may have contributed to the lack of other effects of alcohol use or psychological distress on voice preference.

The qualitative data collected during the study suggested that many fathers considered the messages were acceptable and important for new fathers to receive. While fathers indicated that the text messages made them reflect on their own alcohol consumption levels, some fathers reported they would have benefited from alcohol text messages during their transition to fatherhood. Some high-risk drinkers stated they would have benefited from alcohol text messages at the time they were new fathers to raise awareness of their drinking. Presenting the text messages as questions is a feasible way to encourage self-reflection and autonomy, which is an initial step towards behaviour change for this group. Qualitative data also reinforced that messages were perceived as being more important if they linked alcohol-related harm to the capacity to parent.

The use of the child’s voice appears to have had the desired effect of linking men to their role as a father. Although some fathers did not like the messages in the child’s voice, most responded positively to this approach. Fathers reported that the child voice reminded them that they were dealing with a real person, which made them feel more involved. Some fathers thought that the ‘hey dad’ approach would raise awareness in younger men that were about to become fathers. Many fathers said they lacked awareness of their own drinking behaviour when they had their first child. The fathers who were most enthusiastic about the messages in the child voice also described themselves as being heavy drinkers or ex-drinkers.

In contrast to the positive findings regarding message importance, fathers were more ambivalent about seeking further information prompted by these messages. The lack of effect may relate to the fact that the hypertext links were hypothetical, so they could not imagine what further information they might be accessing. The study also enrolled a high proportion of university-educated fathers that may account for lower response ratings to press on a link for further information as this group may be more aware of alcohol-related harms. However, despite the lower ratings of a hypothetical link in the survey, the qualitative data supported the approach of participants being sent additional information. Participants discussed that being presented with methods to access further information may work well for them.

The qualitative data also allowed exploration of messages this group of fathers considered important to send to prospective fathers to reduce risky drinking. Interestingly, the fathers who drank at high-risk levels or were ex-drinkers had the strongest ideas about alcohol to send to new fathers. They suggested messages directed questions at new fathers about the ‘sort of father they’d want to be’ and suggested ways to moderate their drinking. Fathers who drank at low- to moderate-risk levels suggested messages regarding personal physical and psychological wellbeing, including fitness, obesity, sleep and depression from excessive alcohol consumption. These findings support the concept that alcohol text messages may need to be tailored according to the levels of men’s alcohol consumption.

In addition, these results provide insight into the father’s attitudes about consuming alcohol at recommended levels and their reported drinking behaviour. Data collected during the survey phase showed fathers drinking at higher risk levels considered it less important to drink according to alcohol guidelines and found it more difficult to drink according to recommended levels. This is interesting as most fathers in this study (65%) drank at hazardous levels, which is considerably higher than the national average for males regarding risk of alcohol-related harms in the short term (26%) or lifetime risk (18%) (Australian Institute of Health and Welfare 2014). No specific strategies were employed to recruit a sample with current hazardous alcohol consumption.

Strengths of the current study indicate the feasibility of piloting text messages to fathers around alcohol use and parenting. Message development for the study was underpinned by a Motivational Interviewing framework and the Stages of Change process and guided by an expert group selected for their backgrounds in alcohol treatment and research fields. There are also several limitations to the study, including the use of a small convenience sample and the inclusion of a small proportion of men who had recently become fathers. This selected group of fathers may not be a representative of the broader population of fathers, limiting the generalisability of findings. This study oversampled men considered at-risk drinkers, and most of the fathers were from a higher socio-economic group with a significant proportion having completed tertiary education. This group may be more responsive to behaviour change and more able to give feedback (Elwood 2010; Li and Powdthavee 2015; Ross and Mirowsky 1999). Limitations also include having text messages presented in a survey platform rather than delivered to mobile phones as an actual SMS text message and the use of hypothetical rather than actual hyperlinks.

Future research using the actual format of text messaging and hyperlinks would result in a more accurate estimate of acceptability of the messages and information-seeking behaviour. Text messages and hyperlinks to information tailored to the fathers’ drinking levels could help increase the acceptability of the messages and link pressing behaviour. The AUDIT C and the motivational rulers could be used as assessment tools to deliver messages that align with the father’s drinking behaviour and readiness to change.

Results of this study indicate the feasibility of using alcohol text as a brief intervention to reduce alcohol-related harm in paternal populations. This study established that fathers recruited to the study rated the alcohol text messages developed for this research as important. Data supported the feasibility of using motivational interviewing techniques to increase the salience of the text messages. In addition, fathers indicated that text messages were a highly acceptable format to deliver information about the benefits of reducing risky drinking during the perinatal period. Results suggest that a text message support program for young men transitioning to fatherhood is not only a good initiative, but also a much-needed one. While further research is required to validate the findings, results suggest that motivational text messages could be a cost-effective intervention to address alcohol-related harms in paternal populations.

‘All procedures followed were in accordance with the Ethical standards of the responsible committee on human experimentation (institutional and national) and with the Helsinki declaration of 1975, as revised in 200 (5). Informed consent was obtained from all patients for being included in this study.’
